# The Link between Type III Reg and STAT3-Associated Cytokines in Inflamed Colonic Tissues

**DOI:** 10.1155/2019/7859460

**Published:** 2019-11-06

**Authors:** Xin Xu, Hirokazu Fukui, Ying Ran, Xuan Wang, Yoshihito Inoue, Nobuhiko Ebisudani, Heihachiro Nishimura, Toshihiko Tomita, Tadayuki Oshima, Jiro Watari, Hiroshi Kiyama, Hiroto Miwa

**Affiliations:** ^1^Division of Gastroenterology, Department of Internal Medicine, Hyogo College of Medicine, Nishinomiya, Japan; ^2^Department of Gastroenterology and Hepatology, Tianjin Medical University General Hospital, Tianjin, China; ^3^Department of Functional Anatomy and Neuroscience, Nagoya University Graduate School of Medicine, Nagoya, Japan

## Abstract

Reg (regenerating gene) family proteins are known to be overexpressed in gastrointestinal (GI) tissues under conditions of inflammation. However, the pathophysiological significance of Reg family protein overexpression and its regulation is still unclear. In the present study, we investigated the profile of *Reg* family gene expression in a colitis model and focused on the regulation of Reg III*β* and III*γ*, which are overexpressed in inflamed colonic mucosa. C57BL/6 mice were administered 2% dextran sulfate sodium (DSS) in drinking water for five days, and their colonic tissues were investigated histopathologically at interval for up to 12 weeks. Gene expression of the *Reg* family and cytokines (*IL-6*, *IL-17*, and *IL-22*) was evaluated by real-time RT-PCR, and Reg III*β*/*γ* expression was examined by immunohistochemistry. The effects of cytokines on STAT3 phosphorylation and HIP/PAP (type III REG) expression in Caco2 and HCT116 cells were examined by Western blot analysis. Among *Reg* family genes, *Reg IIIβ* and *IIIγ* were alternatively overexpressed in the colonic tissues of mice with DSS-induced colitis. The expression of STAT3-associated *cytokines* (*IL-6*, *IL-17*, and *IL-22*) was also significantly increased in those tissues, being significantly correlated with that of *Reg IIIβ/γ*. STAT3 phosphorylation and HIP/PAP expression were significantly enhanced in Caco2 cells upon stimulation with IL-6, IL-17, and IL-22. In HCT116 cells, those enhancements were also observed by IL-6 and IL-22 stimulations but not IL-17. The link between type III Reg and STAT3-associated cytokines appears to play a pivotal role in the pathophysiology of DSS-induced colitis.

## 1. Introduction

The *regenerating gene* (*Reg*) was first discovered in regenerating rat pancreatic islets [1], and since then, many *Reg*-related genes constituting a multigene family (types I–IV) have been isolated [2–5]. We and others have previously reported that Reg family proteins are involved in the pathophysiology of gastrointestinal (GI) inflammatory diseases such as *H. pylori*-associated gastritis [6], NSAID-induced GI injuries [7], and inflammatory bowel disease [8–10]. In terms of function, we have clarified that Reg I*α* protein plays a role in tissue regeneration as mitogenic and/or antiapoptotic factor [11, 12], and other Reg family proteins likely have similar roles in inflamed tissues [13–16]. These findings strongly suggest that Reg family proteins are involved in the regeneration of GI tissues that have been injured by inflammation. However, it is still unclear how these proteins with similar function act cooperatively and/or independently in specific GI inflammatory diseases and how Reg family proteins are regulated in such diseases.

Ulcerative colitis is a chronic inflammatory disease characterized by diffuse mucosal inflammation in the colorectum although its pathophysiology has remained largely unclear. Interestingly, comprehensive analyses by several groups have suggested that the expression of *Reg* family genes is distinctly upregulated in the colonic epithelium in UC [17–19], implying a role in the pathophysiology of UC. Indeed, among Reg family proteins, it has been suggested that type III Reg might have a potentially protective effect against colitis [20, 21] and that its effects may be modulated by interaction between type III Reg proteins and the mucosal immune system [22, 23]. These findings suggest that the molecules associated with the mucosal immune system play a pivotal role in the regulation of Reg family protein induction in inflamed colonic tissues, although the mechanism is not yet fully clear. Here, we investigated the profiles of *Reg* family gene expression in a dextran sulfate sodium- (DSS-) induced colitis model, focusing on the regulation of type III Reg in the inflamed colonic tissues.

## 2. Materials and Methods

### 2.1. Animal Model

C57BL/6 mice (eight-week-old females) were used in this study. All the mice were maintained under specific pathogen-free conditions and allowed free access to food and water. The mice were administered 2% dextran sulfate sodium (DSS; molecular weight 36,000–50,000; ICN Biomedicals Inc., Aorano, OH, USA) in drinking water for five days as previously described [24]. Their colonic tissues were removed at various time points, cut open along the longitudinal axis, and fixed in neutral aqueous phosphate-buffered 10% formalin for histological examinations. This animal experiment was performed with the approval of the Animal Use and Care Committee at Hyogo College of Medicine.

### 2.2. Histological Evaluation

Histological evaluation was performed using the tissue sections that were cut perpendicularly to the surface and stained with hematoxylin and eosin. The degree of inflammatory cell infiltration in the colon was scored on a scale of 0 to 3 as follows [24]: 0, normal; 1, inflammatory cell infiltration into the mucosal layer; 2, up to the submucosal layer; and 3, beyond the submucosal layer. The depth of tissue damage in the colon was scored on a scale of 0 to 4 as follows: 0, none; 1, mucosa; 2, submucosa; 3, muscularis propria; and 4, serosa [7]. The histological damage score was evaluated as the sum of those scores for all of the slides of each mouse, and the results were averaged.

### 2.3. Immunohistochemistry

Immunohistochemical staining for Reg III*β* and Reg III*γ* was performed with an Envision Kit (Dako, Kyoto, Japan) as previously described [25], using anti-Reg III*β* antibody (dilution; 1 : 500; gift by Prof. Kiyama) and anti-Reg III*γ* antibody (dilution; 1 : 500; gift by Prof. Kiyama). The immunohistochemical reliability had been confirmed in the nerve system and the intestine in the previous works [26–28]. In brief, the rehydrated sections were treated by microwave heating for 20 min in 1x Dako REAL Target Retrieval Solution (Dako Denmark, Glostrup, Denmark) and then preincubated with 0.3% H_2_O_2_ in methanol for 20 min at room temperature to quench endogenous peroxidase activity. Then, the sections were incubated with primary antibodies for 60 min at room temperature, washed in PBS, and incubated with horseradish peroxidase-conjugated secondary antibody for 30 min. The slides were visualized by 3,3′-diaminobenzidine tetrahydrochloride with 0.05% H_2_O_2_ for 3 min and then counterstained with Mayer's hematoxylin.

### 2.4. Cell Culture and Reagents

Recombinant human IL-6, IL-17, and IL-22 were purchased from R&D Systems (Minneapolis, MN, USA). Anti-human HIP/PAP (REG type III) antibody was purchased from Novus Biologicals (Littleton, CO, USA). Anti-STAT3 and anti-phospho-specific STAT3 (Tyr705) antibodies were purchased from Cell Signaling Technology (Beverly, MA, USA). Anti-*β*-actin antibody was purchased from Sigma.

Human intestinal epithelial cell line Caco2 and HCT116 cells were cultured in RPMI 1640 medium (Invitrogen, Carlsbad, CA, USA) with 10% fetal bovine serum (Biowest, Nuaillé, France) in a humidified incubator at 37°C with an atmosphere of 5% CO_2_. As mentioned in figure legends, the cells were treated with recombinant cytokines at indicated concentrations, respectively.

### 2.5. Western Blot Analysis

Western blot analyses were performed using each primary antibody as previously described [29]. After treatment with or without reagents, cells were lysed in protein extraction buffer. Protein extract (20 *μ*g) was fractionated by SDS-polyacrylamide gel electrophoresis and transferred to a polyvinylidene difluoride membrane. The membrane was incubated with a primary antibody and then with a peroxidase-conjugated secondary antibody. Proteins were detected using an enhanced chemiluminescence system (Amersham Biosciences, Buckinghamshire, UK). ImageJ software (NIH) was used for quantification of intensities of target bands. The staining intensity of *β*-actin was set as the internal control. The value in the individual test was expressed as fold of target protein/*β*-actin in the standard group.

### 2.6. Real-Time RT-PCR

Total RNA was isolated from GI tissues and Caco2 cells with TRIzol reagent (Invitrogen, Carlsbad, CA). Four micrograms of total RNA was reverse-transcribed using oligo-dT primer (Applied Biosystems, Branchburg, NJ), and real-time RT-PCR was performed using 7900H Fast Real-Time RT-PCR System (Applied Biosystems) as previously reported [30]. The set of primers used were shown in Table 1. Real-time RT-PCR assays were carried out with 200 ng of RNA equivalent cDNA, SYBR Green Master Mix (Applied Biosystems), and 500 nmol/l gene-specific primers. The PCR cycling conditions were 95°C for 15 s and 60°C for 60 s. The intensity of the fluorescent dye was determined, and the expression levels of target genes mRNA were normalized to those of *GAPDH* mRNA.

### 2.7. Statistical Analysis

All values were expressed as the mean ± SE. Significance of differences between two animal groups was analyzed by Mann–Whitney *U*-test. Correlations between two parameters were assessed by linear regression analysis. Differences were considered to be significant at *P* < 0.05.

## 3. Results

### 3.1. Histological Features of DSS-Induced Colitis in Mice

**Figure 1:**
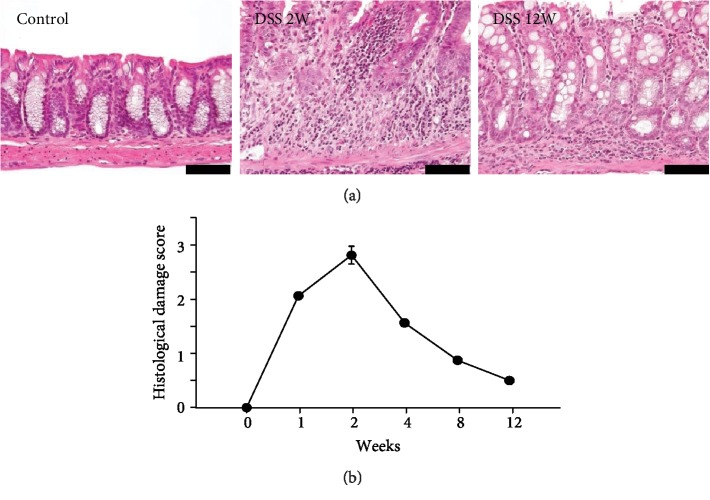
Histological evaluation of colonic tissue in mice with DSS-induced colitis. (a) Colonic tissues in the acute phase (2 weeks after DSS treatment) and healed phase (12 weeks after) and in controls. Bars indicating 50 *μ*m. (b) Histological damage score in colonic tissue of mice with DSS-induced colitis.

DSS treatment induced strong infiltration of inflammatory cells into the colonic mucosa and/or muscular layer (Figure 1(a)). In the acute phase, severe mucosal damage or ulcer formation was observed in some of the experimental mice. The severity of inflammatory cell infiltration peaked at 2 weeks after DSS induction (Figure 1(b)). Thereafter, in the resolution phase, the inflammatory cell infiltration gradually declined but persisted at a very weak level (Figure 1(b)).

### 3.2. Changes in Reg Family Gene Expression in Colonic Tissue of Mice with DSS-Induced Colitis

**Figure 2:**
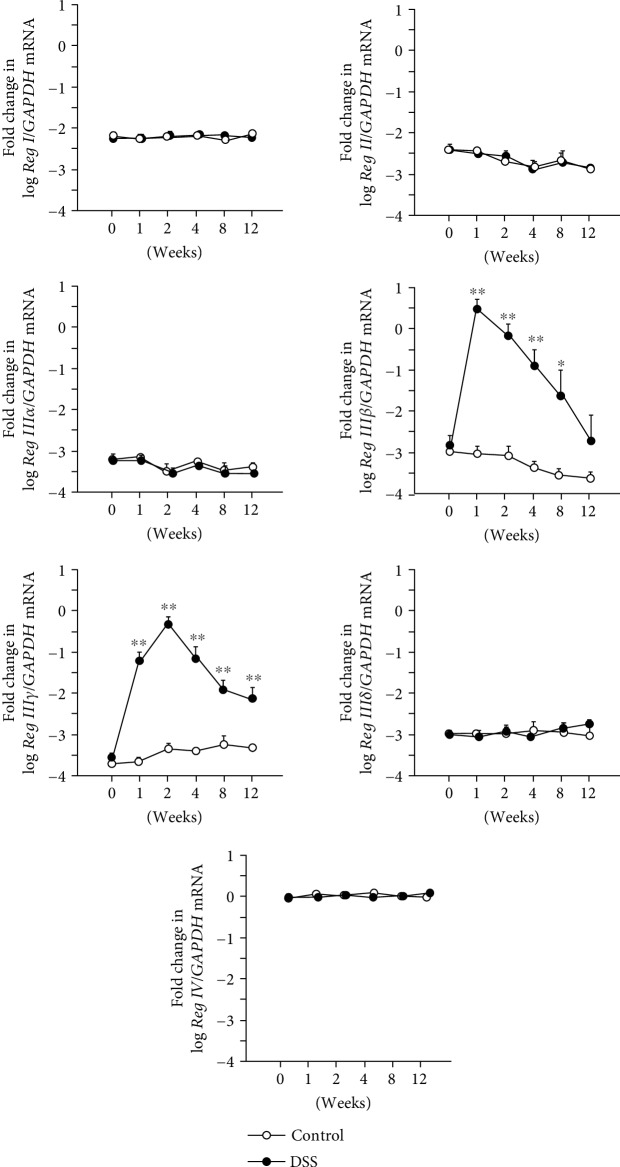
Expression of *Reg* family mRNA in colonic tissue of mice with DSS-induced colitis. Results are expressed as the mean ± SE. Significantly greater than in controls at the same time point: ^∗^*P* < 0.05 and ^∗∗^*P* < 0.01.

We examined changes in *Reg* family gene expression in the colonic tissue of mice at various time points after the induction of DSS colitis (Figure 2). The expression of mRNA for *Reg IIIβ* and *Reg IIIγ* was markedly upregulated in mice with DSS treatment relative to controls. The expression of *Reg IIIβ* peaked at 1 week after DSS treatment and gradually decreased but remained significantly elevated between 1 and 8 weeks after DSS treatment. The expression of *Reg IIIγ* also peaked at 2 weeks after DSS treatment and declined gradually thereafter. However, Reg III*γ* expression was sustained at a significantly high level until the end of the experimental period (Figure 2).

On the other hand, the levels of expression of mRNAs for *Reg I*, *Reg II*, *Reg IIIα*, and *Reg IIIδ* were not altered by the DSS treatment during the experimental period. As reported previously [7], the expression of *Reg IV* was basically high in the colon relative to that of the other *Reg* family genes. However, under the present experimental conditions, the expression of *Reg IV* mRNA did not increase as dramatically as that of *Reg IIIβ* or *IIIγ* (Figure 2).

### 3.3. Relationship between Histology and Reg III*β*/*γ* Expression in DSS-Induced Colitis

**Figure 3:**
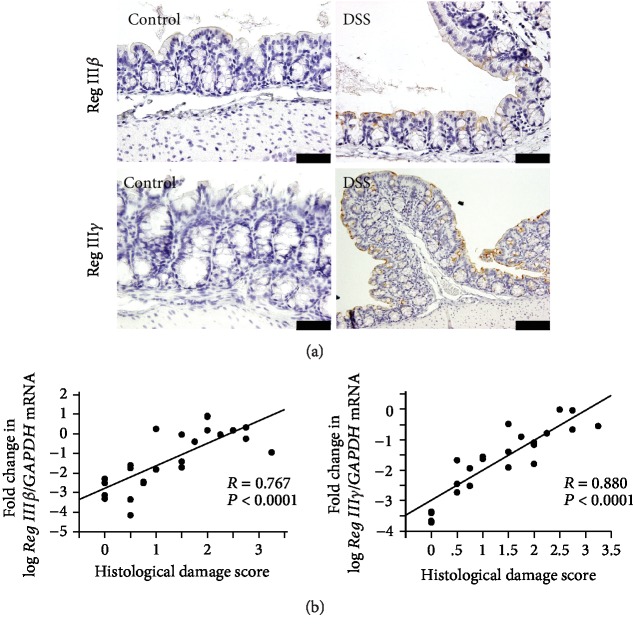
Expression of Reg III*β*/*γ* and its correlation with histological damage score in colonic tissue with DSS-induced colitis. (a) Immunostaining of Reg III*β*/*γ* in the colonic mucosa of mice with DSS-induced colitis and in controls. Bars indicating 50 *μ*m. (b) Correlation between expression of *Reg IIIβ/γ* mRNA and histological damage scores in colonic tissue. Results are expressed as the mean ± SE.

As shown in Figure 3(a), the expression of Reg III*β* and III*γ* proteins was upregulated in the colonic epithelial cells of mice after DSS treatment relative to the untreated controls.

We then investigated the correlation between *Reg IIIβ/γ* expression and histological damage score (Figure 3(b)). The expression of both *Reg IIIβ* and *Reg IIIγ* showed a significantly strong correlation with the histological damage score, suggesting a link with colonic inflammatory injury.

### 3.4. Expression of Cytokines in Colonic Tissue of Mice with DSS-Induced Colitis

**Figure 4:**
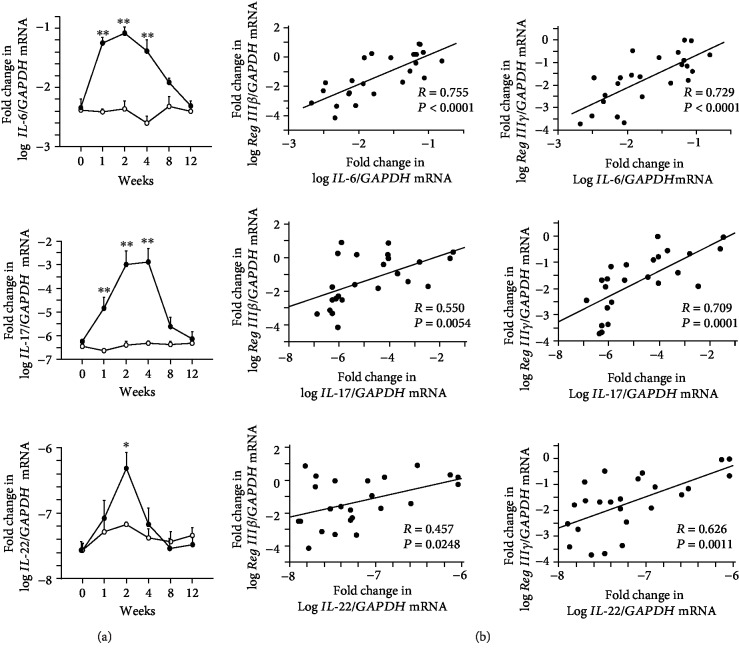
Expression of cytokines and correlation with *Reg IIIβ/γ* expression in colonic tissue of mice with DSS-induced colitis. (a) Expression of *IL-6*, *IL-17*, and *IL-22* mRNA in colonic tissue of mice with DSS-induced colitis and in controls. (b) Correlation between the expression of mRNA for *IL-6*, *IL-17*, and *IL-22* and that of *Reg IIIβ/γ* in the colonic tissue. Results are expressed as the mean ± SE. Significantly greater than in controls at the same time point: ^∗^*P* < 0.05 and ^∗∗^*P* < 0.01.

Previous studies of *Reg I* or *Reg type III* gene expression have suggested that IL-6 and Th17-producing cytokines (IL-17 and/or IL-22) may be key regulators of *Reg* family genes under inflammatory condition [8, 31–34]. Therefore, we investigated the expression of those cytokines in mice with DSS-induced colitis in relation to *Reg IIIβ/γ* expression. The expression of IL-6 and IL-17 mRNA was significantly elevated in tissues of mice with colitis from 1 to 4 weeks after DSS treatment (Figure 4(a)). IL-22 expression was significantly elevated at 2 weeks after DSS treatment. Linear regression analysis showed that *Reg IIIβ/γ* expression was positively correlated with IL-6, IL-17, and IL-22 expressions in the inflamed colonic tissues (Figure 4(b)).

### 3.5. Induction of Human HIP/PAP by Cytokine Stimulation in Caco2 and HCT116 Cells

**Figure 5:**
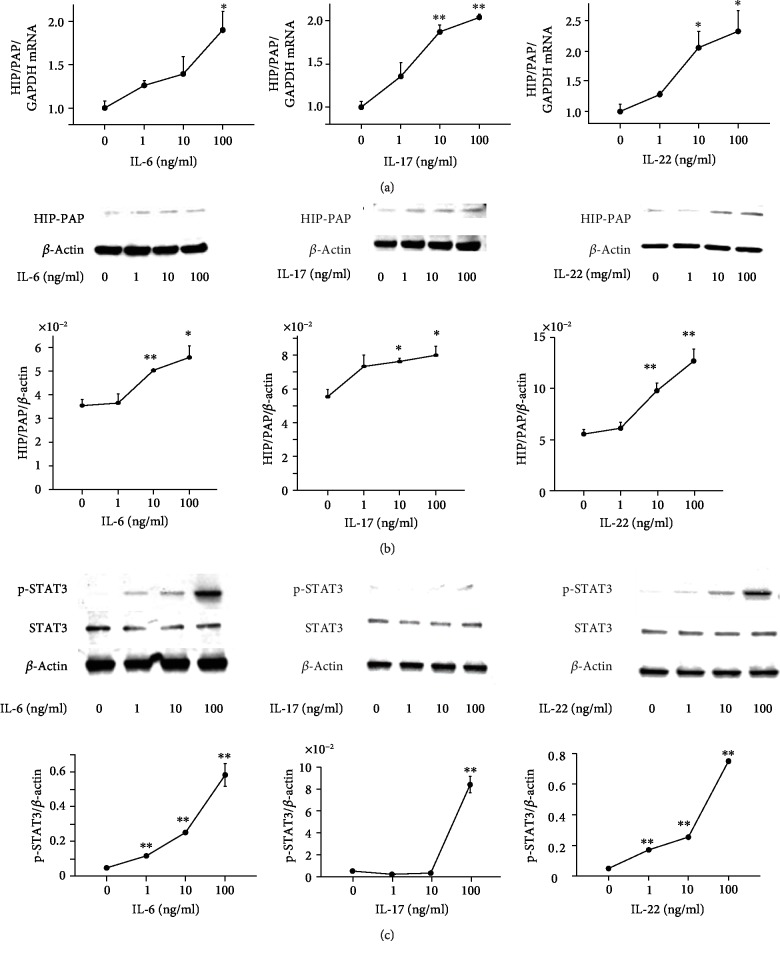
Effect of cytokines on the expression of human HIP/PAP in Caco2 cells. (a) Dose-dependent effect of cytokines (IL-6, IL-17, and IL-22) on *HIP/PAP* mRNA expression in Caco2 cells. Caco2 cells (2 × 10^5^) were cultured in 6-well plates for 24 h and then treated with cytokines (IL-6, IL-17, and IL-22) at the indicated concentrations for 24 h. (b) Dose-dependent effects of cytokines (IL-6, IL-17A, and IL-22) on HIP/PAP protein expression in Caco2 cells. (c) Dose-dependent effects of cytokines (IL-6, IL-17, and IL-22) on phosphorylation of STAT3 in Caco2 cells. Caco2 cells (2 × 10^5^) were cultured in 6-well plates for 24 h and then treated with cytokines (IL-6, IL-17, and IL-22) at the indicated concentrations for 30 min. Results are expressed as the mean ± SE. Significantly greater than in controls at the same time point: ^∗^*P* < 0.05 and ^∗∗^*P* < 0.01.

Human HIP/PAP (hepatocarcinoma-intestine-pancreas/pancreatitis-associated protein) and REG III are classified into type III REG gene and have 85% homology in amino acids sequences [35]. In this study, we examined the effect of cytokines (IL-6, IL-17, and IL-22) on the expression of p-STAT3 and human HIP/PAP in Caco2 cells in vitro. *HIP/PAP* mRNA expression was dose-dependently enhanced by IL-6, IL-17, and IL-22, respectively (Figure 5(a)). In accordance with the results of real-time RT-PCR analyses, the expression of HIP/PAP protein was significantly enhanced by stimulation with IL-6, IL-17, and IL-22 (Figure 5(b)). Moreover, we confirmed that the enhancement of HIP/PAP expression by these cytokines was associated with activated phosphorylation of STAT3 (Figure 5(c)).

**Figure 6:**
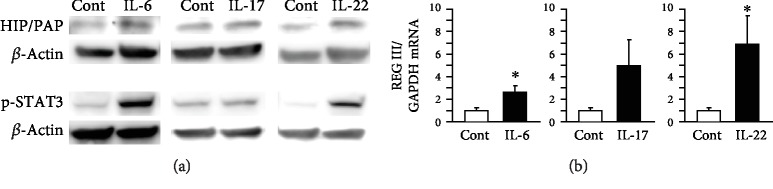
(a) Effect of cytokines on STAT3 phosphorylation and human HIP/PAP expression in HCT116 cells. HCT116 cells (2 × 10^5^) were cultured in 6-well plates for 24 h and then treated with cytokines (IL-6, IL-17, and IL-22) at 100 ng/ml for 24 h and 30 min to examine HIP/PAP expression and STAT3 phosphorylation, respectively. (b) Effect of cytokines on REG III expression. HCT116 cells were stimulated by cytokines at 100 ng/ml for 24 h (*n* = 4). Results are expressed as the mean ± SE. Significantly greater than in controls: ^∗^*P* < 0.05.

The same experiment was carried out using another colon cancer cells (HCT116) (Figure 6). IL-6 and IL-22 stimulations enhanced STAT3 phosphorylation and HIP/PAP expression (Figure 6(a)). On the other hand, IL-17 treatment showed no effects on STAT3 phosphorylation nor HIP/PAP expression in HCT116 cell. As for REG III (Figure 6(b)), its gene expression level was significantly enhanced by IL-6 and IL-22 stimulations. Similar tendency was found in HCT116 cell by IL-17 stimulation (*p* = 0.08) but not statistically significant.

## 4. Discussion

It has been reported that most genes in the human *REG* family (*REG I*, *III*, and *IV*) are overexpressed in inflammatory bowel diseases [36]. In the present study, we investigated the expression profile of *Reg* family genes in DSS-induced colitis as a model of UC. In mice, seven *Reg* family genes have been isolated [2–5, 37], and here, we clarified that *Reg IIIβ/γ*, but not other members of the family, were specifically overexpressed in inflamed colonic tissues. Although it is unclear why the expression of other *REG* family genes was not changed in mouse DSS-induced colitis, unlike the situation in human inflammatory bowel diseases, the difference in species may be partly responsible. The *Reg* family gene profile (i.e., expression intensity and distribution) in the human GI tract has not been investigated comprehensively; however, the expression of REG I*α*, I*β*, III, and IV proteins is detectable in normal colonic epithelial cells by immunohistochemistry [9, 19, 36, 38]. We have previously demonstrated that each gene in the *Reg* family shows predominance in expressional intensity and distribution in the mouse GI tract [7]. In the mouse colon, the expression of *Reg IV* is the strongest, whereas that of *Reg I*, *Reg IIIα/β/γ/δ* is much weaker and *Reg II* is almost undetectable [7]. Therefore, we had expected that *Reg IV* might be predominantly overexpressed in colitis. However, *Reg IIIβ/γ*, whose expression is the strongest in the small intestine under normal conditions, was specifically overexpressed in DSS-induced colitis. Thus, although *REG* family genes may be commonly upregulated in human inflamed GI tissues, their expression appears to be alternatively upregulated, at least in this experimental colitis model.

Here, we demonstrated that Reg III*β* and III*γ* are expressed in colonic epithelial cells and that their gene expression is significantly correlated with the degree of histological damage to colonic tissue, similar to the situation for REG I*α* in human UC [8]. Studies of the mechanism responsible for regulating the expression of *Reg* family genes have shown that cytokines and growth factors play a critical role in this respect [9, 31, 32, 39]. This seems to be reasonable, as cytokines and growth factors are produced abundantly in damaged GI tissues. However, little information is available on the transcriptional factors responsible for the promoter activities of *Reg* family genes. In this connection, we have previously shown that cytokine-associated STAT3 can bind to, and activate, the promoter of the *REG Iα* gene in gastric and colon cancer cells [31, 39]. Other studies have clarified the presence of cytokine (IL-6 and IL-22)-responsive elements in the promoter regions of *REG Iα* and *Iβ* and *HIP/PAP* in pancreatic or colon cancer cells [32, 40], although it remained unclear whether STAT3 and/or other transcriptional factors actually bind to these elements. However, the present findings at least suggest that STAT3-associated cytokines play a pivotal role in the induction of *REG I* and *type III REG* expression. Indeed, it was noteworthy that STAT3-associated cytokines (IL-6, IL-22, and IL-17) were upregulated in the colonic tissues of mice with DSS-induced inflammation and that their expression was significantly correlated with that of both Reg III*β* and III*γ*. Furthermore, we reconfirmed that those STAT3-associated cytokines stimulate the production of HIP/PAP protein through activation of STAT3 phosphorylation *in vitro*. However, we have to describe that detailed analyses of *HIP/PAP* gene promoter are still remained unclear in further studies.

What is the significance of Reg III*β*/*γ* expression in DSS-induced colitis? Similar to REG I, II, and IV [9, 11–13, 16], type III REG proteins are known to act as mitogenic and/or antiapoptotic factors [14, 15], implying a role in the prevention of tissue damage and/or the regeneration of injured tissues. Ogawa et al. have demonstrated that not only DSS-induced colitis but also the presence of commensal gut microbiota is important for the induction of Reg III*β*/*γ* expression in the colon [10]. Although the data are not conclusive, it is interesting to consider whether bacteria affect Reg III*β*/*γ* expression in GI epithelial cells directly or indirectly via activation of cytokine-producing immune cells. Reg family proteins have a characteristic C-type lectin structure [41], and much attention has recently been paid to the possibility that these proteins may act as antimicrobial factors in the GI tract and skin [22, 23, 42, 43]. Together, the data strongly suggest that overexpression of Reg III*β*/*γ* in colonic tissues of mice with DSS-induced colitis represents a protective and/or reparative mucosal defense mechanism.

In summary, among genes of the *Reg* family, *Reg IIIβ* and *IIIγ* were alternatively overexpressed in the colonic epithelial cells of mice with DSS-induced colitis. In this experimental model, the expression of STAT3-associated cytokines such as IL-6, IL-17, and IL-22 was significantly increased, and these cytokines clearly upregulated HIP/PAP expression via activation of STAT-3 phosphorylation *in vitro*. Furthermore, we showed that the *in vivo* expression of *Reg IIIβ/γ* was significantly correlated with that of STAT3-associated cytokines in this model of DSS-induced colitis. These findings suggest that STAT3-associated cytokine/type III Reg axis plays a pivotal role in the pathophysiology of not only the acute phase but also the healing process of colitis. Although the accumulated data strongly suggest that type III Reg proteins have a protective role against colitis, future studies will need to clarify the mechanism by which these proteins operate in colitis.

## 5. Conclusions

Type III Reg protein linked to STAT3-associated cytokine stimulation plays a pivotal role in the pathophysiology of DSS-induced colitis in mice.

## Figures and Tables

**Table 1:** Primers for real-time RT-PCR analysis.

Gene	Direction	Primer sequence
*Mouse-Reg I*	Forward	5′-GAACGCCTACTTCATCCTGC-3′
	Reverse	5′-GATGGCAGGTCTTCTTCAGC-3′
*Mouse-Reg II*	Forward	5′-GATCAGCATGGCTCAGAACA-3′
	Reverse	5′-TCTTCAGCTACCTGGCCTTG-3′
*Mouse-Reg IIIα*	Forward	5′-CTCAGGACATCTCGTGTCTATTCT-3′
	Reverse	5′-AGTGACCACGGTTGACAGTAGAG-3′
*Mouse-Reg IIIβ*	Forward	5′-TCCCAGGCTTATGGCTCCTA-3′
	Reverse	5′-GCAGGCCAGTTCTGCATCA-3′
*Mouse-Reg IIIγ*	Forward	5′-TTCCTGTCCTCCATGATCAAAA-3′
	Reverse	5′-CATCCACCTCTGTTGGGTTCA-3′
*Mouse-Reg IIIδ*	Forward	5′-TGGAACCACAGACCTGGGCTA-3′
	Reverse	5′-GAGCAGAAATGCCAGGTGTC-3′
*Mouse-Reg IV*	Forward	5′-CGCTGAGATGAACCCCAAG-3′
	Reverse	5′-TGAGAGGGAAGTGGGAAGAG-3′
*Mouse-IL-6*	Forward	5′-CCAGTTGCCTTCTTGGGACT-3′
	Reverse	5′-GGTCTGTTGGGAGTGGTATCC-3′
*Mouse-IL-17A*	Forward	5′-GACTCTCCACCGCAATG-3′
	Reverse	5′-CGGGTCTCTGTTTAGGCT-3′
*Mouse-IL-22*	Forward	5′-TCCGAGGAGTCAGTGCTAA-3′
	Reverse	5′-AGAACGTCTTCCAGGGTGAA-3′
*Mouse-GAPDH*	Forward	5′-GGAGAAACCTGCCAAGTATG-3′
	Reverse	5′-TGGGAGTTGCTGTTGAAGTC-3′
*Human-HIP/PAP*	Forward	5′-AGAGAATATTCGCTTAATTCC-3′
	Reverse	5′-AATGAAGAGACTGAAATGACA-3′
*Human-REG III*	Forward	5′-GAATATTCTCCCCAAACTG-3′
	Reverse	5′-GAGAAAAGCCTGAAATGAAG-3′
*Human-GAPDH*	Forward	5′-GAGTCAACGGATTTGGTCGT-3′
	Reverse	5′-TTGATTTTGGAGGGATCTCG-3′

## Data Availability

The datasets used and/or analyzed during the current study are available from the corresponding author on reasonable request.
